# Students’ perceived restorativeness of university environment: the validation of the Rest@U scale

**DOI:** 10.3389/fpsyg.2024.1348483

**Published:** 2024-07-12

**Authors:** Elisa Menardo, Margherita Brondino, Ottavia Damian, Marco Lezcano, Camilla Marossi, Margherita Pasini

**Affiliations:** Department of Human Science, University of Verona, Verona, Veneto, Italy

**Keywords:** restorativeness, academia, students, validation, environment

## Abstract

University students are likely to encounter mental health issues throughout their educational journey. Among the various factors that can impact students’ wellbeing, the physical environment can potentially restore cognitive, physiological, and emotional resources, thereby enhancing academic performance, and overall quality of life, while reducing feelings of stress and depression. The Perceived Restorativeness Scale is the most commonly used tool to assess the level of restorativeness derived from the educational physical environment. However, a tailored measure could be a more psychometrically suitable approach to capture the context-specific characteristics of university environments for academic students. This study aimed to validate an instrument that can accurately evaluate university spaces to measure the perceived restorativeness of university students. A total sample of 685 students from two Italian universities participated in the evaluation of the psychometric properties of the Restorativeness at University scale (Rest@US), consisting of 13 items divided into four dimensions: fascination, being-away, scope, and coherence. The hypothesised four-factor model (being-away, fascination, scope, and coherence) demonstrated excellent fit indices in both the calibration and validation samples and was invariant for sex. The scale demonstrates good reliability. Furthermore, criterion validity has been confirmed, highlighting that, in a theoretically consistent manner, the perceived restorativeness of university physical environments from the point of view of students and its dimensions were negatively correlated with techno-overload and study-related workload and positively correlated with perceived performance and psycho-physical wellbeing.

## 1 Introduction

Administrators at higher learning institutions are concerned about their students’ psychological wellbeing as university students often encounter and experience many demanding and stressful situations (Yusli et al., 2021). Indeed, university students’ mental health is an important public health issue (Sheldon et al., 2021) because they have a higher risk of developing cognitive problems such as stress, anxiety, and depression than the general population (Ibrahim et al., 2013; Brenneisen Mayer et al., 2016). The systematic review proposed by Sheldon et al. (2021) reveals that, while studying at university, 25% of students experience depression and 14% of students experience outcomes related to suicide. A World Health Organization (WHO) survey of 1,572 university students interviewed also identified that one-fifth (20.3%) exhibited DSM disorders (Auerbach et al., 2016). Sheldon et al. (2021) also observed a higher rate of depression in student populations than the general population, which has a prevalence of 12.9% (Lim et al., 2018). The narrative synthesis proposed by the authors and the meta-analysis on risk factors highlighted several key determinants of mental health problems among university students, encompassing individual, interpersonal, and systemic factors (Sheldon et al., 2021). Considering the issue from a systemic approach, factors such as academic pressures, cost-related stress, and difficulties associated with the social environment have certainly been considered. A meta-synthesis by Hazell et al. (2020) highlighted that university environments can influence the mental wellbeing of doctoral students, emphasising that the problem spans across all levels of higher education. These results underscore the importance of addressing the issues from a contextual perspective, especially from a preventive standpoint. This involves considering both organisational and cultural issues, identifying guidelines for best practices, and exploring potential protective contextual factors. This also includes the physical environment in which students spend their time at the university; therefore, understanding the design insights from the research can help using the biophilic design properly on university campuses.

### 1.1 The role of the physical environment in restoration

Students need opportunities to restore cognitive and emotional resources, and the physical environment plays a role in students’ overall experience. For this reason, it may also contribute to the restoration of cognitive and emotional resources. Restorative environments, designed to promote relaxation, stress reduction, and overall wellbeing, are increasingly considered key settings for promoting health (Frumkin, 2001; Bratman et al., 2019). The restorativeness of an environment could be defined as its ability to promote (not only allow) the recovery of resources (biological, cognitive, psychological, and social) in an individual (Hartig, 2004). This can be considered a source of psychological restoration, which refers to activities or environments that help individuals recover from mental fatigue, stress, and cognitive overload. Engaging in restorative experiences is linked to several positive health outcomes. Natural environments are considered the most restorative environment (for a review, see Berto, 2014; McMahan and Estes, 2015; Ohly et al., 2016; Menardo et al., 2019). However, in recent years, researchers’ attention has shifted to build environments where people spend most of their time, both in their private lives and during work or study experience. The concept of restorative environments is closely connected to the notion of stress. Individuals adopt coping strategies to protect themselves from potential stressors, i.e., ways to deal with stressful situations. Among these strategies, exposure to natural environments has been identified as a promoter of both stress reduction and recovery of cognitive abilities that may have declined due to mental fatigue. The most frequently cited theories in this regard are the biophilia hypothesis (Wilson, 1984), attention restoration theory (ART, Kaplan and Kaplan, 1989; Kaplan, 1995), and the stress reduction theory (SRT, Ulrich, 1983; Ulrich et al., 1991), which suggest that contact with nature influences both cognitive recovery and wellbeing. Many studies have supported these positive effects of natural environments (Menardo et al., 2019). According to the literature (e.g., Kaplan, 1995; Korpela and Hartig, 1996; Pasini et al., 2014), some environmental features successfully enhance the quality of restoration in individuals. According to Kaplan’s paradigm (1995), frequently used in research in this field, there are four regenerative factors: fascination, which refers to how an environment might attract the involuntary attention of a person; being-away, which refers to how an environment causes a person to feel freed from everyday demands and obligations; extent, a characteristic composed by coherence, which refers to how an environment is perceived as organised or not, and scope that refers to how an environment offers the possibility of exploration; compatibility which refers to the correspondence between the characteristics of an environment and expectations of a person.

While research extensively emphasises the positive effects of natural environments, an equally significant line of inquiry explores the restorative potential of built spaces within the “biophilic design” framework (Ulrich, 1983; Kaplan, 1995; Berto et al., 2015). Biophilic design, rooted in the concept of biophilia, posits that humans possess a genetic predisposition to love nature (Wilson, 1984). This approach focuses on the crucial features that built environments must incorporate to foster healthy spaces. Authors such as Chawla (2002), Colucci-Gray et al. (2006), and Kellert (2002) underscore the importance of establishing a connection with nature, especially in childhood, to shape meaningful relationships and positive environmental sentiments. This instinctual connection, called biophilia (Wilson, 1984), is inherent to the human species. Stephen R. Kellert, a pioneer in biophilic design, advocates for its widespread adoption to innovate in shaping spaces. Biophilic design aims to integrate individuals with nature through architectural elements, materials, and psychological responses to the environment (Kellert et al., 2011). While direct contact with nature is crucial, as emphasised in attention restoration theory (ART) and stress reduction theory (SRT), the biophilic approach surpasses the mere inclusion of elements such as green walls or natural light. In his research, Kellert (2008) conceptualised biophilic design through six elements: environmental features, natural shapes and forms, natural patterns and processes, light and space, place-based relationships, and evolved human–nature relationships. These six categories are detailed with over 70 biophilic design attributes. As exposure to nature, the biophilic design could reduce stress and anxiety (Gray and Birrell, 2014; Roskams and Haynes, 2020) and enhance students’ wellbeing and quality of life (Hipp et al., 2016; Gulwadi et al., 2019; Payne et al., 2020; Yusli et al., 2021). Different research on biophilic design cites studies in restorative environments and claims that biophilic elements could improve the restorativeness of the built environment (e.g., Joye, 2007; Gray and Birrell, 2014; Ryan et al., 2014). However, if the effect of natural elements (e.g., plants and nature view from windows) on stress and cognitive functioning has been largely investigated (Gritzka et al., 2020; Aydogan and Cerone, 2021), few research studies have explored the effect of the others biophilic elements (Gillis and Gatersleben, 2015). As an example of the application of this framework in designing a workplace environment, Pasini et al. (2021) conducted a research project on biophilic design’s impact on workplace wellbeing. Through the participatory design of the work environment, and using biophilic design principles, the team aimed to reduce stressors, resulting in improved perceived restorativeness. This improvement correlated with enhanced quality in specific workplace elements, such as light, air, acoustics, natural views, and destress areas. In particular, the study found a positive link between the improved quality of these elements and increased job satisfaction and work engagement. In conclusion, this approach can provide interesting insights for the design of work environments or university campuses, so that these structures can become places where wellbeing and sustainability, alongside performance, are priorities.

### 1.2 The effect of restorative environments on several outcomes for university students

It has been shown that a restorative physical environment plays an important role in enhancing students’ wellbeing and quality of life (Hipp et al., 2016; Gulwadi et al., 2019; Payne et al., 2020; Yusli et al., 2021). The World Health Organization (WHO) defines quality of life as an individual’s perception of their position in life in the context of the culture and value systems in which they live, and concerning their goals, expectations, standards, and concerns. It is a broad-ranging concept that encompasses various aspects of an individual’s wellbeing, including their physical health, psychological state, level of independence, social relationships, personal beliefs, and their relationship to their environment. Hipp et al. (2016) used the WHOQOL-BREF, a short version of an instrument developed by the WHO, to evaluate quality of life (QOL) in a sample of university students. The instrument covers four main domains: physical health, psychological health, social relationships, and environment (which include financial resources, safety, access to health services, and the quality of living conditions). Using a cross-sectional research design, they assessed both perceived greenness and perceived restorativeness of university students concerning their university campus, in association with QOL. They found that perceived greenness was correlated with both QOL and perceived restorativeness; moreover, QOL was significantly correlated with perceived restorativeness. The path model showed that perceived restorativeness partially mediates the relationship between greenness and QOL. In a later study, Gulwadi et al. (2019) proposed a similar correlational study involving two university students’ samples (one from a campus in Turkey and one from a campus in the United States). Once again, the study considered the QOL of students in relation not only to the perception of greenness and restorativeness but also to objectively measured greenness using the Normalised Difference Vegetation Index (NDVI). This measurement considered three campus environments (central, building, and overall). The results indicated that the objective measurement of greenness correlates with the subjective one and that this objective measure is a predictor of QOL, both directly and mediated by subjective perceptions of greenness and regenerativeness.

Recent studies have examined the relationship between the physical environment and the wellbeing of university students. For instance, Payne et al. (2020) highlighted a positive correlation between restorativeness and students’ wellbeing, as well as a statistically significant negative relationship between restorativeness and distress. Using a one-way ANCOVA, the authors verified that students in the experimental group, who spent time in a natural environment, showed a significant decrease in stress levels, although not in burnout or quality of life compared to the control group. In this way, the hypothesised beneficial predicting effect of natural environments on stress and quality of life was only partially confirmed. However, after comparing the single pre- and post-intervention results for each variable, a statistically significant difference between the values of burnout and quality of life has been noted between the two groups. Participants in the experimental group experienced a greater average decrease in stress and burnout levels than those in the control group. This second result leads the authors to conclude that natural environments have a beneficial effect. A cross-sectional study conducted by Yusli et al. (2021) assessed the association between the perceived restorativeness of university students and psychological wellbeing, considering the four dimensions of the attention restoration theory (ART): fascination, being-away, extension, and compatibility. The study also examined the view of nature from the window as a moderating variable. The results demonstrated a positive relationship between three of the four regenerative factors with wellbeing (excluding the extent factor). Furthermore, the data analysis emphasised the moderating effect of the view of nature from the window on the relationship between these three factors and wellbeing.

Examining the effects of greenery and restorative environments on cognitive performance, it is possible to identify studies that specifically focus on the university student population. Studente et al. (2016) used a between-subjects experimental research design to examine the impact of exposure to greenery on visual and verbal creativity in a group of university students. These students were randomly assigned to three groups corresponding to three conditions (two experimental and one control groups). The results showed that having the opportunity to see elements of nature, such as plants inside the room or a view of nature from the window, alongside the green colour of the sheet on which they had to respond, enhanced visual creativity, although it did not affect verbal creativity. According to the attention restoration theory (ART), having the opportunity to experience a natural environment has restorative effects on cognitive aspects, such as working memory, after a cognitive depletion task. In a study conducted by van Oordt et al. (2022), a group of university students observed a digitally presented nature scene, an urban scene, or no specific scene after completing a task that depleted working memory capacity (WMC). They then performed a digit span task to assess the restoration of WMC. The results showed that performance was better for those who had observed natural scenes compared to the other two groups. Some studies have shown that natural environments can improve students’ self-discipline and concentration abilities. Taylor et al. (2002) compared and demonstrated that students who have access to natural views, plants, and the colour green in a classroom show greater visual creativity than students in a classroom with blinds drawn to block the view of natural settings. Mason et al. (2022) reviewed 14 studies in the existing literature that report investigations involving students at university in a study with short exposure to nature during a study day. The review shows that in 12 of the aforementioned studies, benefits on cognitive processes emerge, in terms of directed attention restoration from mental fatigue and improvement in tasks that evaluate executive functions, due to contact with nature in natural and campus environments. Furthermore, various evidence shows how natural features in a school’s environment promote better academic performance (Matsuoka, 2010; Hodson and Sander, 2017; Li et al., 2019). For example, concerning the exposure to trees as a natural scene, Matsuoka (2010) found that trees and shrubs were positively correlated with academic performance, such as standardised test scores, graduation rates, percentages of students planning to attend a 4-year college, and fewer occurrences of criminal behaviour, while exposure to lawn spaces showed a negative relationship. Li et al. (2019) highlighted a positive correlation between performance and the density of trees near school buildings, and Hodson and Sander (2017) found a significant positive relationship between tree cover in school environments and reading performance, suggesting that initiatives aimed at increasing tree cover in student environments could support academic success.

### 1.3 Perceived restorativeness in the university students’ context

In the context of universities, different research has shown that nature exposure, design, and campus resources can impact restorativeness. For example, objective (i.e., the amount of green space) and perceived greenness were positively associated with the student perceptions of restorativeness (Hipp et al., 2016; Gulwadi et al., 2019). Moreover, windows that overlook nature can enhance the restorative potential of university spaces (Wang et al., 2018; Yusli et al., 2021), such as the presence of water features, planting flowers, and scattered trees (Wang et al., 2018; Lu and Fu, 2019). In addition, the possibility of regularly engaging in walking (Chou and Hung, 2021) or the duration of the visit (Du et al., 2022) in a natural environment increases perceived restorativeness. Finally, natural indoor elements, such as large murals, could enhance the restorative power of university indoor spaces (Felsten, 2009). These studies, except one (Felsten, 2009), investigated the whole university environment and not specific spaces. This is probably linked to the characteristics of students’ routines. Indeed, students attend different spaces on the university campus to participate in different activities (e.g., attending lectures, individual study, and meetings with other students and/or professors). To investigate the university environment, the most used scale (e.g., Hipp et al., 2016; Gulwadi et al., 2019) was the Perceived Restorativeness Scale (PRS) developed by Hartig et al. (1997). This scale consists of 26 items that assess four key components of perceived restorativeness: fascination, i.e., the extent to which the environment is perceived as interesting, intriguing, or capturing attention; being-away, the degree to which the environment allows individuals to feel a sense of detachment from their usual concerns and obligations; extent, which evaluates the feeling that the environment provides a sense of scope and the opportunity to explore; compatibility, i.e., the perception that the environment is congruent with personal inclinations, preferences, or activities. The PRS is relatively straightforward to administer and analyse, making it accessible to researchers with diverse backgrounds and interests. This clear connection with the ART theoretical framework, joined with the ease of use in different contexts, contributes to its widespread adoption. This scale has been used to evaluate the perceived restorativeness in many different locations, such as educational, hospital, residential, and working spaces. Even though the use of the PRS managed to lead to statistically significant results, other groups of researchers decided to use other research tools or to adapt the PRS to evaluate the perceived restorativeness of campuses related to their research. An example of variation can be found in the study by Felsten (2009). In this study, the researcher made students rate their perceived restorativeness using only one item for each of the PRS components (being-away, fascination, extent, and compatibility). In addition, a final item was added to assess the overall perceived restorativeness of the students. In other cases, different scales have been applied. In the experiment of Payne et al. (2020), the Restorative State Scale (RSS; van den Berg et al., 2014) was used to evaluate how the past experiences of students in a natural environment had a positive effect on their mental health when asked them to recall to their mind that experience. Another example of the use of a different scale is the Short-version Revised Restoration Scale (SRRS; Han, 2003). In one of the two experiments reported in the study of Wang et al. (2018), the researchers used the SRRS to evaluate the essential attributes of a mentally restorative landscape on a Chinese university campus. However, these scales (i.e., PRS and RSS) were developed to investigate natural (not built) environments, so these tools could not be suitable for the university environment as the opportunities for restoration vary according to the type of activities that an individual performs. It is worth noting that, in both the study and work environments, the formulation of certain items may fail to capture the profound sense of the dimensions of restorativeness, according to the theoretical approach of the attention restoration theory (ART) underlying the mentioned scale. For example, statements such as “I feel far away from everyday concerns” or “This place is a refuge from the demands of my daily life” (two examples of items from the B-A dimension in the PRS) may be totally inappropriate when applied to work or study contexts. As known, the consequences of an inadequate measuring instrument can be detrimental to the validity of research because they amplify the possibility of measurement errors. The fact that some items from scales traditionally used to measure the perceived restorativeness cannot be employed due to their inadequacy in capturing the specific dimension does not imply that these places cannot still possess characteristics that make them regenerative.

Other studies (Lu and Fu, 2019; Chou and Hung, 2021; Du et al., 2022) developed specific *ad hoc* questionnaires to evaluate the perceived restorativeness in campus locations. If in some cases (Chou and Hung, 2021; Du et al., 2022) these questionnaires were built through different items, containing parts of scales such as the Attention Recovery and Reflection (Staats et al., 2003) or the Recovery Component Scale (Laumann et al., 2001), in others (Lu and Fu, 2019) self-reporting measures, based on the attention restoration theory (ART), in which the perception that the environment is congruent with personal inclinations, preferences, or activities, have been made and used for evaluations. However, no scale has already been validated from a psychometric point of view that allows us to profile the restorative abilities of the university space. Brondino et al. (2023) have proposed a tool to assess workers’ perception of restorativeness concerning the physical work environment. This process involved delving into the meaning of dimensions described in the attention restoration theory (ART) from the perspective of workers. This undertaking led to the development of a scale, the Rest@Work scale, which also served as the foundation for creating a specific tool for assessing the restorativeness of university study environments. The objective of this study was indeed to take the first step towards validating a tool that is specific for evaluating the perceived restorativeness of university spaces.

## 2 Methods

### 2.1 Participants

Two samples of Italian academic students, one for the calibration and one for the validation of the factor structure of the scale, were used in the present study. The first sample was composed of 247 undergraduate students [64% women, mean (sd) age = 22.6 (4.5), age range = 19–56]. In total, 302 students agreed to participate in the survey. Of these, 55 (18%) were excluded: 50 did not complete the scale, and 5 were multivariate outliers. Participants mainly attended a degree course in psychology (51%) or physiotherapy (28%).

The second sample was composed of 400 undergraduate students [70% women, mean (sd) age = 21.3 (5.08), age range = 18–57]. In total, eight multivariate outliers were excluded. Most of the participants were enrolled in a degree course in psychology (81%).

### 2.2 Procedure

Students filled out a battery of questionnaires, including a scale aimed at measuring the perceived restorativeness of places within the university (Rest@U Scale) from the point of view of students, and, only for the validation sample, other measures to test criterion validity, in a controlled situation (e.g., in a room or during a zoom meeting, with the supervision of the research team). Upon accessing the survey on LimeSurvey, students were first presented with detailed instructions about the purpose of the study. This introductory section explained the procedure to complete the survey and emphasised the importance of honest and individual responses without consultation. Before proceeding, students were required to provide informed consent, ensuring that they understood the objectives of the study, their rights as participants, and the confidentiality measures in place. Once informed consent was obtained, participants were directed to the main section of the survey, where they responded to the set of questions. Participation was voluntary and data anonymous. Data collection was done from November to December 2022 for the first sample and from November to December 2023 for the second one. The research was approved by the Ethical Committee of the Department of Human Science of the University of Verona (Prot. number 2022_27).

### 2.3 Development of the restorativeness at university scale

The Rest@US was adapted by the authors from the restorativeness-at-work scale (Rest@WS, Brondino et al., 2023) to investigate the perceived restorativeness of the university’s physical environment. The instruction was adapted as follows: “Thinking about the university you attended the most in the last week, carefully read each of the following sentences and then evaluate on a scale from 0 to 10 how much each statement corresponds to your experience in this place.” We chose to ask for an evaluation of the university environment in general, rather than specific spaces because student activities are diverse (e.g., attending lectures, individual study, and meetings with other students and/or professors), and these activities are carried out in various university spaces. Therefore, we were interested in building a tool capable of assessing the restorativeness of the physical environment of the university as a whole. The Rest@US comprises 13 items, each rated on an 11-point Likert scale (not at all–very much). Items investigated the following dimensions of the ART: fascination (three items), being-away (three items), scope (three items), and coherence (four items). A pool of four experts examined the wording of the items adapted to the university context about content validity. All the items were judged adequate. After that, a pilot study with 38 undergraduate students [84% female, mean (SD) age = 19.9 (1.7), age range = 18–28] was run to investigate the quality of the items (descriptive statistics, correlation, and discrimination index). The aim was to verify the appropriateness of the response scale and the ability of each item to discriminate different levels of the construct. Students filled out a battery of questionnaires, including R@US (see the Appendix for the italian version of the item), in a classroom with the supervision of one of the authors. Participation was voluntary and data anonymous, and informed consent was filled in by all participants. Descriptive statistics of items are presented in Table 1. All items were normally distributed except item #7 (“The place where I study is messy”), which has a leptokurtic and negative symmetrical distribution.

**Table 1:** Descriptives of items from the pilot study (*n* = 38).

Id	Dimension	Item	Mean	Standard deviation	Min	Max	Asymmetry	Kurtosis
R@US 1	COH	The place where I study makes me mentally tired because of how it is structured*	7.05	2.40	2	10	−0.41	−0.92
R@US 2	FA	In the place where I study, my attention can be attracted by many interesting things *(*e.g.*, furnishings, a beautiful view…)*	3.18	2.30	0	10	0.85	0.83
R@US 3	B-A	I am able to take a little break to think or do something pleasant at the place where I study	4.39	2.69	0	10	0.33	−0.88
R@US 4	COH	In the place where I study the spaces are well organised	6.45	2.26	1	10	−0.59	−0.16
R@US 5	COH	In the place where I study, I easily find the things I need to work	5.95	2.38	2	10	0.02	−0.92
R@US 6	B-A	The place where I study has elements that allow me to relax my mind from time to time *(*e.g.*, plants, or a poster of a nice place etc)*	2.58	2.83	0	10	0.86	−0.34
R@US 7	COH	The place where I study is messy*	8.71	2.09	1	10	−2.49	6.49
R@US 8	B-A	The place where I study is structured in such a way that if I need I can stay focused or if I want I can let my mind wander *(for example looking out the window)*	6.42	3.18	0	10	−0.37	−1.27
R@US 9	FA	The place where I study has elements or characteristics that stimulate my curiosity	3.87	2.65	0	10	0.54	−0.42
R@US 10	SCO	In the place where I study there are barriers that prevent my eyes wandering (i.e., a small room with few features)*	6.87	2.75	0	10	−0.93	0.06
R@US 11	FA	The place where I study has characteristics that fascinate me	4.29	2.54	0	10	0.19	−0.87
R@US 12	SCO	The place where I study is designed so that I can visually explore space in many directions	4.32	2.60	0	10	0.13	−0.56
R@US 13	SCO	The place where I study is harmoniously structured	5.03	2.54	0	10	0.13	−0.61

COH, coherence; FA, fascination; B-A, being-away; SCO, scope. *means reversed item.

To investigate the discrimination ability of items, the distribution was divided into four using quartiles (except item #7, which was divided into three using tertiles). We used the Mann–Whitney test and the corresponding effect size (r = z/√N) to verify the difference between the lower and the upper group for each item with respect to the correspondence dimension’s score. For all comparisons, the difference was significant with a high effect size (r range = 0.62–0.80), which indicates a high discrimination ability. All items were used in the main study because no one had floor or ceiling effects, and all of them had high discrimination ability.

### 2.4 Other measures

*Workload* was measured by three items from the HSE Management Standards Indicator Tool (Toderi et al., 2013; Balducci et al., 2017), to investigate student mental workload, or “how hard students work.” An example of an item is “I have unreachable deadlines” with a response scale from 1 = Never-almost never to 5 = Always (Cronbach’s α = 0.67).

*Technostress* was detected by the subdimension techno-overload of the TCS Technostress Creator Scale (Ragu-Nathan et al., 2008), which was adapted and translated into Italian by Molino et al. (2020). It was measured with four items, e.g., “I am forced by technology to work much faster,” with a response scale from 1 = Totally disagree to 7 = Totally agree (Cronbach’s α = 0.72).

*Student psycho-physical* wellbeing was measured with the General Health Questionnaire (GHQ-12) using the Italian version of 12 items (Piccinelli et al., 1993) on a response scale from 1 = Much less than to 4 = Better than usual (e.g., “Have you recently been able to concentrate on whatever you are doing?”; Cronbach’s α = 0.87).

Finally, *perceived academic performance* was measured with a single *ad hoc* item: “In your opinion, expressing it through a percentage (from 0 to 100%), to what extent have you succeeded in achieving the goals you set yourself over the past year with regard to your study activity?”

Furthermore, some *socio-demographic* variables were collected, specifically sex, age, and the attended degree programme.

### 2.5 Data analysis

Preliminary analyses were performed following the suggestions of Tabachnick and Fidell (2013). First, we checked for the normal distribution of each Rest@US item and for the presence of univariate outliers (± 3.29 standard deviation from the group mean). Second, based on the Mahalanobis distance, we searched for the presence of multivariate outliers and checked for normal multivariate distribution (Mardia’s test).

The theoretical four-factor structure was checked through confirmatory factor analysis (CFA) using the R package Lavaan (Rosseel, 2012) in two samples (calibration and validation sample). We used the diagonally weighted least squares (DWLS), which is specifically designed for ordinal data and does not assume normally distributed variables (Li, 2016). The overall goodness of fit was evaluated using the chi-square statistic (χ^2^), the Comparative Fit Index (CFI; Bentler, 1990), the root mean square error of approximation (RMSEA; Steiger, 1990), and the standardised root mean square residual (SRMR; Bentler, 1995). Cutoffs that are usually used to verify the goodness of fit (Bentler and Bonett, 1980; Schermelleh-engel, 2003) are not adequate when the DWLS estimator is used (Xia and Yang, 2019; Groskurth et al., 2023). For this reason, we computed a tailored cutoff following the equation-based approach developed by Groskurth et al. (2023). In this approach, the cutoff is predicted by regression formulae based on the computed coefficient, empirical data, and study characteristics. The calibration sample value higher than 0.983 for CFI, lower than 0.072 for RMSEA, and lower than 0.126 for SRMR suggests a reasonable fit. The validation sample value higher than 0.972 for CFI, lower than 0.061 for RMSEA, and lower than 0.094 for SRMR suggests a reasonable fit. To improve the fit of the model, we looked for modification indices (MIs) for each specified model, factor loading, and r squared of item.

In both the samples, the four-factor structure was compared with alternative nested models, which were theoretically plausible: (A1) one obtained by collapsing the four factors into one factor, (A2) one obtained by collapsing the factors in two factors (fascination + being-away and coherence + scope), and (A3) one in which the four first-order factors aggregate into a second-order factor. To this aim, Δχ2, ΔCFI, and ΔRMSEA were used as fit indices. To indicate that the null hypothesis of equivalence should be rejected (i.e., that the four-factor structure model had a better fit than the alternative models), a significant Δχ2 is required. Moreover, a value of ΔCFI (which is less affected by sample size) higher than 0.01 and a ΔRMSEA value higher than 0.015 indicated a deterioration of fit (Cheung and Rensvold, 2002).

The appropriateness of the CFA sample size was verified by calculating the statistical power of the model (MacCallum et al., 1996) using the R package “semPower” (Moshagen and Erdfelder, 2016). Following the *post-hoc* approach of MacCallum et al. (1996), we fixed the effect size (RMSEA) level to 0.5 and the alpha level to 0.05.

As the Rest@US is a multidimensional scale, we used McDonald’s ω coefficient (McDonald, 1999) implemented in the R package semTools (Jorgensen, 2022).

In the validation sample, we also assessed measurement invariance across sex, analysing configural invariance, metric invariance, and scalar and strict invariance. In accordance with Chen’s criteria (Chen, 2007), invariance was confirmed if CFI changes less than 0.01, RMSEA less than 0.015, and SRMR less or equal to 0.030 (less or equal to 0.010 for assessing scalar invariance). Finally, criterion validity was assessed by analysing the bivariate correlations between the perceived restorativeness of the university’s physical environment and the single dimensions of the scale, with some outcome variables (techno-overload, study-related workload, perceived academic performance, and psycho-physical wellbeing).

## 3 Results

We first conducted some preliminary analyses to assess data quality and distribution. In both samples, no univariate outliers were found. However, we found and excluded five multivariate outliers in the calibration sample and five in the validation sample.

In both samples, all observed variables were univariate normally distributed (skewness and kurtosis were between approximately −1 and + 1) with again the exception of item 7 (“My university campus is messy”), which reported, respectively, in both the samples skewness = −2.49/1.22 and kurtosis = 6.49/1.07 (see Table 2). The calculated Mardia’s indices for the calibration and the validation sample were equal to 226.00 and 212.80, respectively. They were higher than the critical value of 195, suggesting that the data were not multivariate normally distributed in both samples.

**Table 2:** Descriptives of items from calibration and validation studies (*n* = 247/ 400).

Id	Dimension	Item	Mean	Standard deviation	Min	Max	Asymmetry	Kurtosis
R@US 1	COH	The place where I study makes me mentally tired because of how it is structured*	4.65/ 3.20	3.44/ 2.48	0/0	10/10	0.02/0.55	−1.39/ −0.47
R@US 2	FA	In the place where I study. my attention can be attracted by many interesting things *(*e.g.*, furnishings. a beautiful view…)*	3.30/ 4.12	3.00/ 2.46	0/0	10/10	0.60/0.31	−0.78/ −0.74
R@US 3	B-A	I am able to take a little break to think or do something pleasant at the place where I study	5.21/ 6.07	3.39/ 2.52	0/0	10/10	−0.09/ -0.59	−1.35/ −0.43
R@US 4	COH	In the place where I study the spaces are well organised	4.85/ 6.05	2.76/ 2.20	0/0	10/10	0.02/ -0.58	−0.93/ −0.13
R@US 5	COH	In the place where I study. I easily find the things I need to work	4.60/ 5.31	2.74/ 2.32	0/0	10/10	0.12/ -0.17	−0.93/ −0.65
R@US 6	B-A	The place where I study has elements that allow me to relax my mind from time to time *(*e.g.*, plants, or a poster of a nice place etc)*	2.63/ 3.12	3.04/ 2.78	0/0	10/10	0.93/0.62	−0.40/ −0.80
R@US 7	COH	The place where I study is messy*	3.19/ 1.97	2.48/ 2.00	0/0	10/10	0.43/ 1.21	−0.58/ 1.05
R@US 8	B-A	The place where I study is structured in such a way that if I need I can stay focused or if I want I can let my mind wander *(for example looking out the window)*	5.18/ 6.72	3.03/ 2.33	0/0	10/10	−0.05/ -0.69	−1.03/0.03
R@US 9	FA	The place where I study has elements or characteristics that stimulate my curiosity	3.59/ 5.03	2.90/ 2.54	0/0	10/10	0.50/ -0.21	−0.81/ −0.76
R@US 10	SCO	In the place where I study there are barriers that prevent my eyes wandering (i.e., a small room with few features)*	4.71/ 3.28	2.95/ 2.45	0/0	10/10	−0.02/0.48	−0.94/ −0.72
R@US 11	FA	The place where I study has characteristics that fascinate me	3.59/ 5.26	2.92/ 2.47	0/0	10/10	0.42/ -0.31	−0.99/ −0.79
R@US 12	SCO	The place where I study is designed so that I can visually explore space in many directions	4.15/ 5.40	2.87/ 2.45	0/0	10/10	0.30/ -0.26	−0.83/ −0.66
R@US 13	SCO	The place where I study is harmoniously structured	4.03/ 5.26	2.53/ 2.18	0/0	10/10	0.30/ -0.28	−0.64/ −0.29

COH, coherence. FA, fascination. B-A, being-away. SCO, scope. *means reversed item.

After preliminary analyses, we conducted a confirmatory factor analysis first on the calibration sample and later on the validation sample.

**Figure 1:**
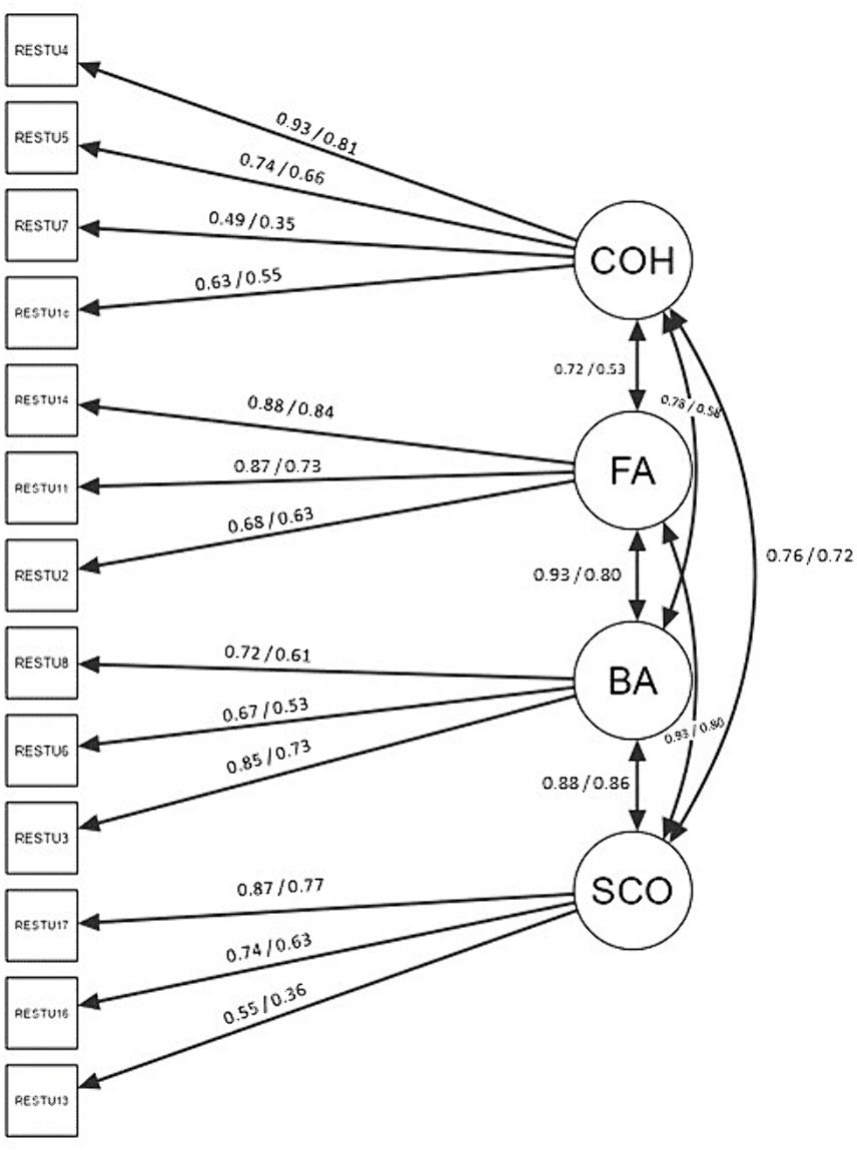
Factorial model of the hypothesised model of restorativeness dimensions. The digits represent standardised factor loadings and latent correlations on the calibration and the validation sample. All the presented estimates were statistically significant (*p* < 0.001).

In the calibration sample, the hypothesised four-factor model with 13 items showed an excellent fit (χ^2^ = 54.42, *p* = 0.68; CFI = 1.000; RMSEA = 0.000, 90% C.I. = 0.000, 0.032; SRMR = 0.047; explained variance = 56%). As shown in Figure 1, the factor loadings ranged from 0.49 to 0.93 (mean and median = 0.74) and were all statistically significant (*p* < 0.001). The interfactor correlations ranged from 0.72 to 0.93. The confidence interval for each interfactor correlation did not include the value of 1, indicating that the four factors were separate.

Cronbach’s alpha of the four dimensions was at least good, specifically 0.79 for coherence, 0.85 for fascination, 0.79 for being-away, and 0.75 for scope. The internal coherence of the total scale was very good (McDonald’s ω = 0.93). The statistical power of the model was good (0.86).

As shown in Table 3, Δχ^2^ suggested that the hypothesised four-factor model is better than the alternative models obtained by collapsing the four factors into one (A1) or two factors (A2 and A3). However, ΔCFI and ΔRMSEA confirmed a significant deterioration of fit only for the one-factor model (A1) and the two-factor model (A2). On the contrary, the hierarchical model (A3) was equivalent to the four-factor model.

**Table 3:** Fit indices of the four-factor model and alternative models (calibration sample).

	CFI	TLI	RMSEA	SRMR	*χ^2^*_(*df*)_	Δ*χ^2^*_(Δ*df*)_	ΔCFI	ΔRMSEA	ΔSRMR
4-factor	1.000	1.002	0.000 (0.000–0.032)	0.047	53.42 _(59)_	–	–	–	
A1 - 1-factor	0.992	0.991	0.046 (0.026–0.064)	0.064	98.87_(65)_	45.44_(1)_***	−0.008	0.046	0.027
A2 - 2 factor (FA + BA) & (SCO+COH)	0.994	0.993	0.039 (0.015–0.058)	0.061	88.27_(64)*_	34.85_(1)_***	−0.006	0.039	0.014
A3.4-factor hierarchical	1.000	1.001	0.000 (0.000–0.033)	0.049	56.53 _(61)_	3.11_(1)_	0.00	0.00	0.002

COH, coherence. FA, fascination. B-A, being-away. SCO, scope. *p < 0.05, **p < 0.01, ***p < 0.001.

On the validation sample, the confirmatory factor analyses confirmed the goodness of the hypothesised four-factor model (χ^2^ = 135.34, p < 0.001; CFI = 0.972; RMSEA = 0.056, 90% C.I. = 0.044, 0.069; SRMR = 0.062; explained variance = 44%; see Table 4). Factor loading and interfactor correlations are reported in the path diagram in Figure 1. Factor loadings were all statistically significant (*p* < 0.001) and ranged from 0.35 to 0.85 (mean and median = 0.64). The interfactor correlations ranged from 0.53 to 0.83. Again, as in the calibration sample, the confidence interval for each interfactor correlation did not include the value of 1, indicating that the four factors were separate.

**Table 4:** Results of confirmatory factor analysis and measurement invariance across sex (validation sample).

	CFI	TLI	RMSEA	SRMR	*χ^2^*_(*df*)_	Δ*χ^2^*_(Δ*df*)_	ΔCFI	ΔRMSEA	ΔSRMR
4-factor	0.972	0.963	0.056 (0.040–0.069)	0.062	135.34 _(59)_	–	–	–	
A1. 1-factor	0.938	0.926	0.083 (0.072–0.094)	0.085	244.26_(65)_	108.92_(1)_ ***	−0.034	0.027	0.023
A2. 2-factor (FA+BA) & (SCO + COH)	0.826	0.778	0.116 (0.103-0.129)	0.082	273.00_(43)_	137.66_(1)_ ***	−0.174	0.060	0.020
A3. 4-factor hierarchical	0.967	0.958	0.063 (0.051–0.075)	0.067	157.01 _(61)_	21.67_(1)***_	−0.005	0.007	0.005
MI across Sex									
Configural invariance	0.979	0.973	0.051 (0.035–0.065)	0.071	178.06_(118)_	–	–	–	–
Metric invariance	0.976	0.97	0.053 (0.038–0.067)	0.075	198.43_(127)_	20.37**	−0.003	0.002	0.004
Scalar invariance	0.976	0.973	0.051 (0.036–0.064)	0.072	205.41_(136)_	6.98	0.000	−0.002	−0.003
Strict invariance	0.977	0.976	0.047 (0.033–0.061)	0.078	215.45_(149)_	10.04	0.001	−0.004	0.006

COH, coherence. FA, fascination. B-A, being-away. SCO, scope. *p < 0.05, **p < 0.01, ***p < 0.001.

The reliability of the four dimensions was slightly worse than that of the calibration sample. Cronbach’s alpha was 0.70 for coherence, 0.78 for fascination, 0.65 for being-away, and 0.60 for scope. The internal coherence of the total scale was good (McDonald’s ω = 0.87).

To test invariance across sex, we ran a sequence of gradually more restrictive tests on the parameters of the hypothesised model. The results for configurational, metric, scalar, and strict invariance are all confirmed (see Table 4).

Criterion validity was assessed by analysing Pearson’s bivariate correlations between restorativeness and its dimensions with techno-overload, study-related workload, perceived academic performance, and psycho-physical wellbeing (see Table 5). As theoretically hypothesised, techno-overload and study-related workload were negatively related to restorativeness and all its dimensions. Correlations were all statistically significant apart from being-away and ranged for techno-overload from −0.06 (Fascination) to −0.15 (coherence and restorativeness) and for study-related workload from −0.10 (fascination) to −0.24 (coherence). Performance and psycho-physical wellbeing, coherently, were positively correlated with restorativeness and its dimensions. Performance was more correlated with restorativeness (0.14) and with being-away (0.13). However, the correlations related to coherence (0.10) and scope (0.09) were statistically significant only considering a one-tailed test. For psycho-physical wellbeing, the correlations ranged from −0.02 (fascination) to 0.13 (coherence).

**Table 5:** Pearson’s bivariate correlation between restorativeness, its dimensions, and other constructs (validation sample).

	REST@U (total)	REST@U Coherence	REST@U Being-away	REST@U Fascination	REST@U Scope	Techno-overload	Study-workload	Performance	Psychophysical wellbeing
REST@U (total)	1								
REST@U_coh	0.72***	1							
REST@U_ba	0.80***	0.35***	1						
REST@U_fa	0.81***	0.36***	0.62***	1					
REST@U_sco	0.80***	0.48***	0.56***	0.53***	1				
Techno-overload	−0.15**	−0.15**	−0.12*	−0.06	−0.14**	1			
Study-workload	−0.20***	−0.24***	−0.14**	−0.10*	−0.15**	0.25***	1		
Performance	0.14**	0.10	0.13*	0.12*	0.09	−0.19***	−0.21***	1	
Psychophysical Wellbeing	0.09	0.13**	0.07	−0.02	0.09	−0.23***	−0.29***	0.38***	1

COH, coherence. FA, fascination. B-A, being-away. SCO, scope. *p < 0.05, **p < 0.01.

## 4 Discussion

As shown in the literature and stressed in the introduction of this study, the results about the effects of perceived restorativeness are growing. Perceived restorativeness seems to be linked with positive outcomes, promoting relaxation, reduction of stress, and overall wellbeing (Frumkin, 2001; Bratman et al., 2019). The restorative quality of an environment can be characterised by its capacity to facilitate, not just permit, the replenishment of resources (biological, cognitive, psychological, and social) in an individual (Hartig, 2004). This study arises from the awareness of the need to pay attention to the wellbeing of university students, considering contextual factors, including specifically the physical environment in which students carry out their activities within the university. The literature review has allowed an understanding of the importance of a construct such as restorativeness, i.e., the ability of physical environments to regenerate individuals, both cognitively and in terms of stress recovery. However, examining this literature has revealed a lack of specific attention to the quality of the instrument used to measure this construct, often applying tools uncritically more suitable for assessing natural environments where people spend leisure time, or environments not considering the demands of work or study. While it is not sensible to assume that such physical environments cannot simultaneously be places of engagement and restorative environments, it is crucial to recognise their specificity as environments where people are engaged in activities that involve physical and mental fatigue. The importance of using measurement tools for psychological constructs with excellent psychometric properties is well known, as a first step to ensure the validity of research. This study aimed to identify a valid and reliable measuring instrument specifically designed to assess perceived restorativeness among university students when evaluating the physical environments in which they study at their universities. The identification of the items in this scale is in continuity with the work done to develop the Rest@Work scale, where four dimensions identified as fundamental, starting from ART (fascination, being-away, coherence, and scope), were articulated to deepen the meaning of each dimension with the specificity of what is carried out in the work environment. The research was conducted using a calibration sample, employed to assess construct validity, and a validation sample to confirm the goodness of the model identified in the first step. Additionally, the validation sample was used to evaluate criterion validity by incorporating specific constructs potentially correlated with restorativeness.

The results of confirmatory factor analyses confirmed the hypothesised model, indicating the presence of the four dimensions and a higher order factor identifying restorativeness. This confirmation was held true in both the calibration and validation samples. Furthermore, in the latter, there was also strong criterion validity observed through correlations with other constructs theoretically linked to restorativeness, such as stress (specifically technostress), wellbeing, and performance. From the data analysis, it has been clear that the new items managed to maintain the relationships in the existing literature. From the results, we can observe the presence of a negative correlation between perceived restorativeness and the dimensions of techno-overload and study-related overload. On the contrary, perceived restorativeness has been found to correlate positively with psycho-physical wellbeing. Even if with a slightly different connotation, these results are similar to the one stated by Payne et al. (2020) in their research. In their study, the scholars stated that the restorativeness produced from natural areas and evaluated through the RSS is negatively correlated with distress (burnout and stress) and positively correlated with wellbeing (life satisfaction).

A more relevant conclusion about the usefulness of specific items to evaluate perceived restorativeness can be drawn from the results obtained while looking for positive associations with perceived performance. This result is in line with the ones reported in the Mason et al. (2022) review, where exposure to natural and campus green environments led to better performances in cognitive tests and higher activations of brain areas related to executive functions while performing a task. This association between natural elements and performances does not stop only in the university grades, but it has a broader expansion in other levels of education, such as primary and secondary schools.

Despite the important results obtained from the present research, we can identify a series of limits that must be considered during this discussion. First, the type of population should be considered. First, the limitation pertaining to the conduction of the study within Italian universities poses several implications for the generalisability of the findings. The specificity of cultural, historical, and institutional context, educational structure, and language aspect constrains the results obtained in the Italian-specific context. In summary, while the focus of the study on Italian universities provides valuable insights into a specific context, it also necessitates caution when generalising findings to broader cultural and educational contexts. Researchers should consider these limitations when interpreting the results. To overcome these limitations and to enhance the robustness and applicability of our findings, we are planning to collect data from other countries and from different educational backgrounds to analyse the psychometric properties of this specific scale in different cultural contexts.

Another limitation is related to the research design. In the evaluation of criterion validity for variables such as perceived performance and psycho-physical wellbeing, a longitudinal study would have been more appropriate. Furthermore, in the criterion validity analysis, it should be acknowledged that criterion variables were consistently assessed using self-report scales, leading to the potential issue of common method variance.

## 5 Conclusion

This study can be considered as the first attempt in the creation and evaluation of a context-specific scale to evaluate the perceived restorativeness of the physical environment at the university from the point of view of students. Moreover, one of the aims of this scale is to support future research by giving a wide-use tool adaptable specifically to the various university environments and campuses existing as more studies continue to be conducted in these contexts. The promising results obtained state how specific tools are needed for the research field as they allow the gathering of more reliable and accurate data and, consequently, findings. The lack of literature on specific scales, addressed in the introduction part of this study, concerning the investigation of restorativeness in different types of environments and spaces experienced daily, states the relevance of the results obtained in this study, aimed at developing a specific instrument for this specific potentially demanding physical environment. Therefore, this scale can permit future research to focus on the restorativeness of university spaces. The results of this future research and the application of the scale will be precious for the university to evaluate their spaces, valorising the ones resulting as already restorative and implementing action policies to improve the ones that are not very restorative, also inserting new biophilic features. To conclude, this new path can contribute to the definition of guidelines for the creation of new biophilic and restorative environments for students and universities.

## Data availability statement

The raw data supporting the conclusions of this article will be made available by the authors, without undue reservation.

## Ethics statement

The studies involving humans were approved by Ethical board of the Department of Human Sciences of the University of Verona, Italy. The studies were conducted in accordance with the local legislation and institutional requirements. The participants provided their written informed consent to participate in this study.

## Author contributions

EM: Conceptualization, Data curation, Methodology, Writing – original draft, Formal analysis, Writing – review & editing. MB: Conceptualization, Data curation, Formal analysis, Funding acquisition, Methodology, Project administration, Supervision, Writing – original draft, Writing – review & editing. OD: Data curation, Writing – original draft. ML: Data curation, Writing – original draft. CM: Data curation, Writing – original draft. MP: Conceptualization, Funding acquisition, Methodology, Project administration, Supervision, Writing – original draft, Writing – review & editing.

## Appendix

Italian version of the scale.

Pensando alla sede universitaria che hai frequentato di più nell’ultima settimana leggi attentamente ciascuna delle seguenti frasi e poi valuta su una scala da 0 a 10 quanto ogni affermazione corrisponde alla tua esperienza in questo luogo.

**Table:**

Il luogo in cui studio mi affatica mentalmente per come è strutturato
Nel luogo in cui studio la mia attenzione può essere attratta da tante cose interessanti (es. elementi d’arredo, una bella vista…)
Il luogo in cui studio è anche pensato per potersi prendere una piccola pausa per pensare o fare qualcosa di piacevole
Nel luogo in cui studio gli spazi sono ben organizzati
Nel luogo in cui studio trovo facilmente le cose che mi servono
Il luogo in cui studio, per come è strutturato, ha degli elementi che di tanto in tanto mi permettono di svagarmi con la mente (ad es. ci sono poster di luoghi di vacanza)
Il luogo in cui studio è disordinato
Il luogo in cui studio è strutturato in modo tale che se ho necessità posso stare concentrato o se voglio posso divagare, ad esempio guardando fuori dalla vetrata/finestra
Il luogo in cui studio ha degli elementi o delle caratteristiche che stimolano la mia curiosità
Il mio luogo di studio è fatto in modo che io possa esplorare lo spazio in molte direzioni
Il mio luogo di studio è strutturato in modo armonioso
